# Supplementary material for: Incorporating longitudinal history of risk factors into atherosclerotic cardiovascular disease risk prediction using deep learning
Source: Sci Rep. 2024 Jan 31;14:2554. doi: 10.1038/s41598-024-51685-5 (PMC10830564; doi:10.1038/s41598-024-51685-5)
Supplement: Supplementary file 1 — Supplementary Information. [file 41598_2024_51685_MOESM1_ESM.docx]

**Supplemental Tables and Figures**

**Table 1.** Baseline demographics and clinical characteristics of the participants by cohort.

|  | **ARIC** | **CARDIA** | **FHS** | **FOF** |
| --- | --- | --- | --- | --- |
| N | 8852 | 2306 | 2115 | 2292 |
| Age | 53.98 (5.73) | 45.21 (3.63) | 57.35 (7.10) | 48.94 (9.23) |
| Male | 4931 (55.7%) | 1303 (56.5%) | 1191 (56.3%) | 1194 (52.1%) |
| Black | 2169 (24.5%) | 1072 (46.5%) | 0 (0.0) | 0 (0.0) |
| SBP, mm Hg | 120.34 (18.09) | 115.71 (14.64) | 137.90 (21.31) | 124.04 (16.69) |
| DBP, mm Hg | 73.18 (10.93) | 72.23 (11.14) | 83.18 (10.85) | 79.27 (9.50) |
| Total Cholesterol, mmol/L | 214.57 (41.25) | 185.75 (35.05) | 240.97 (45.51) | 212.65 (41.48) |
| HDL, mmol/L | 51.90 (16.98) | 54.24 (16.67) | 48.32 (14.61) | 51.01 (14.73) |
| Smoker | 2186 (24.7%) | 447 (19.4%) | 865 (40.9%) | 665 (29.0%) |
| Diabetes | 814 (9.2%) | 164 (7.1%) | 121 (5.7%) | 85 (3.7%) |
| Hypertension Treatment | 2549 (28.8%) | 401 (17.4%) | 243 (11.5%) | 375 (16.4%) |
| ASCVD Cases | 1098 (12.4) | 127 (5.5%) | 97 (4.6%) | 406 (17.7%) |

**Table 2**. Distribution of the sex in the training and testing set

| Training | | |
| --- | --- | --- |
|  | Male | Female |
| Other | 34.4% | 38.3% |
| Black | 10.9% | 16.4% |
| Testing | | |
|  | Male | Female |
| Other | 35.0% | 39.1% |
| Black | 9.7% | 16.3% |

**Table 3.** DeepHit Model Results

**Table 3. a)** *DeepHit* model training and testing AUROC.

| **Model** | **Training AUROC** | **Test AUROC** |
| --- | --- | --- |
| *DeepHit* (Cross-sectional) | 0.812 (0.798-0.826) | 0.808 (0.778-0.838) |

**Table 3. b)** *DeepHit* model AUROC in various demographic groups.

| **Categories** | ***DeepHit* AUROC** |
| --- | --- |
| Race & Sex | |
| Other Males | 0.790 (0.744-0.836) |
| Other Females | 0.794 (0.732-0.857) |
| Black Males | 0.823 (0.755-0.890) |
| Black Females | 0.823 (0.753-0.892) |
| Age | |
| ,< 60 years | 0.795 (0.776-0.814) |
| ≥ 60 years | 0.809 (0.778-0.839) |

**Table 4**. *Dynamic-DeepHit* results in specific cohorts. AUCs calculated from specific cohorts within the test dataset.

| **Cohort** | **Cohort Specific Test AUC** |
| --- | --- |
| ARIC | 0.799 (0.767-0.831) |
| CARDIA | 0.867 (0.806-0.927) |
| FHS | 0.731 (0.661-0.802) |
| FOF | 0.755 (0.688-0.822) |
